# Supplementary material for: Organic electrochemical transistors as on-site signal amplifiers for electrochemical aptamer-based sensing
Source: Nat Commun. 2023 Mar 25;14:1665. doi: 10.1038/s41467-023-37402-2 (PMC10039935; doi:10.1038/s41467-023-37402-2)
Supplement: Supplementary file 1 — Supplementary Information [file 41467_2023_37402_MOESM1_ESM.pdf]

Supplementary Information

Organic Electrochemical Transistor as an On-site Signal Amplifier for Electrochemical Aptamer-based Sensing

Xudong Ji[1,2], Xuanyi Lin[3,4,5] and Jonathan Rivnay[1,2]

1. Department of Biomedical Engineering, Northwestern University, Evanston, IL, 60208, USA.
2. Simpson Querrey Institute, Northwestern University, Chicago, IL, 60611, USA.
3. Center for Sleep and Circadian Biology, Northwestern University, Evanston, IL, 60208, USA.
4. Department of Neurobiology, Northwestern University, Evanston, IL, 60208, USA.
5. Department of Psychology, The University of Hong Kong, Pokfulam Road, Hong Kong.

*jrivnay@northwestern.edu

Supplementary Note 1

Derivation of OECT amplification equation: The channel current can be defined by the Ohm's Law as:

$$I_{\mathrm{DS}} = Wde\mu\rho(x)[\mathrm{d}V(x)/\mathrm{d}x] \quad (1)$$

Where W and d are the channel width and thickness, e is elementary charge, μ is hole mobility, $\rho(x)$ is hole density, $V(x)$ is the voltage along the channel. By assuming the linear increase of the Drain/Source voltage along the channel, the independence of mobility on the hole density, the channel current modulation is purely caused by the change of hole density, which results from the gate current induced ion injection. It can be written as:

$$\Delta I_{\mathrm{DS}} = Wde\mu\Delta\rho(x)\frac{V_{\mathrm{DS}}}{L} \quad (2)$$

Where L is the channel length and $\Delta\rho(x)$ is the change of hole density, which can be defined as:

$$\Delta\rho(x) = \frac{\int I_G dt}{eWdL} \quad (3)$$

Combining equation 1~3, the channel current modulation is given by:

$$\Delta I_{\mathrm{DS}} = \frac{\mu V_{\mathrm{DS}}}{L^2}\int I_{\mathrm{G}}\mathrm{dt} \quad (4)$$

Supplementary Note 2

A comparison of the TGF-β_1 sensing with different technique is shown in Supplementary Table 1. Overall, our ref-OECT based E-AB sensor shows much higher sensitivity compared with all other technique, which further demonstrate the amplification property of OECT. While for detection limit, indeed we show higher detection limit, while we think there is room to improve. In terms of comparison with sensors in each individual work, for reference 1 and 2, the device is exactly same as the electrode-based E-AB sensor we have shown. As expected, our ref-OECT based E-AB sensor shows much higher sensitivity and similar detection limit. For reference 3, the device is developed using similar approach as electrode-based E-AB sensor while with a graphene/AuNPs modification of the Au working electrode. This nanomaterial modification enables them to have lower detection limit than us; however, our sensor still outperforms in sensitivity. It shows an opportunity to improve the detection limit of our sensor by using nanomaterials modified working electrode (gate). For reference 4~7, although they show good detection limit, sophisticated sandwiched modification assay is used, and the in-vivo implementation may present additional challenges. Specifically, sensors in reference 4 and 6 use dissolved redox probe in solution, while sensors in reference 5 and 7 use H_2O_2 to react with the material on the sensing electrode. Both dissolved redox probe and H_2O_2 limited their applicability for in-vivo implementation, which is very important as a future direction.

sensing technique	electrode modification	recognition element	sensitivity	detection limit	potential in-vivo application	ref.
SWV	bare Au	aptamer	~42 nA/dec	~1 ng/mL	Y	[1]
SWV	bare Au	aptamer	~42 nA/dec	~1 ng/mL	Y	[2]
SWV	graphene+ AuNPs	aptamer	~2 µA/dec	~0.025 ng/mL	Y	[3]
SWV	bare Au	peptide	143.3 µA/dec	0.011 ng/mL	N	[4]
CA	SWCNT	antibody	332 nA/dec	0.95 pg/mL	N	[5]
EIS	bare Au	antibody	NA	0.57 ng/mL	N	[6]
CA	SWCNT	antibody	~300 nA/dec	1.3 pg/mL	N	[7]
CV/SWV -OECT	bare Au	aptamer	292/290 µA/dec	1 ng/mL	Y	this work

Supplementary Table 1. Summary of the performance of different biosensors for TGF-β_1 sensing

Supplementary Figures

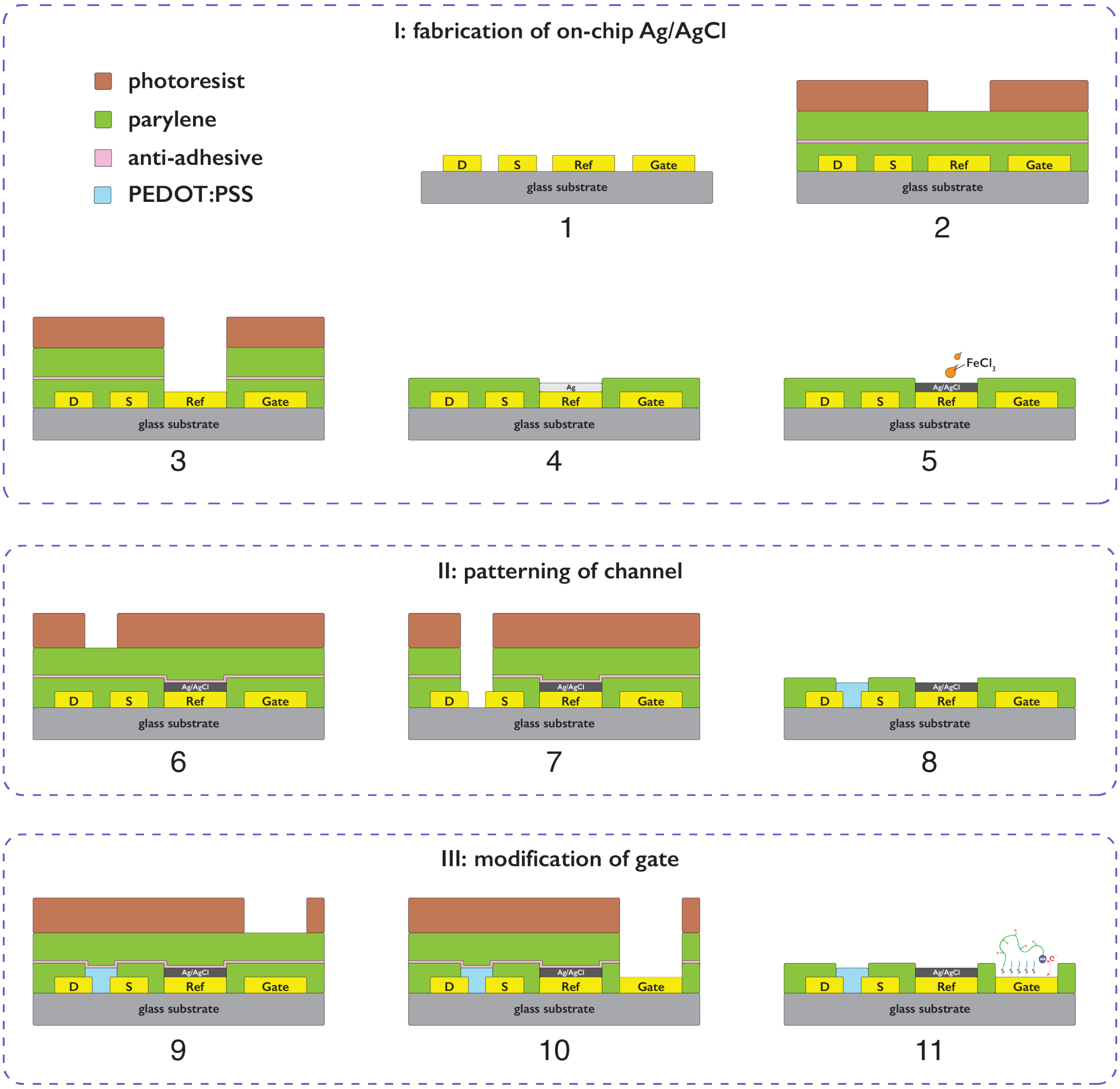

Supplementary Figure 1. Fabrication scheme of the different components in the ref-OECT-based E-AB sensor. Multiple parylene dry peel-off process have been used to separately pattern the on-chip Ag/AgCl reference electrode, PEDOT:PSS channel and the aptamer modified sensing gates.

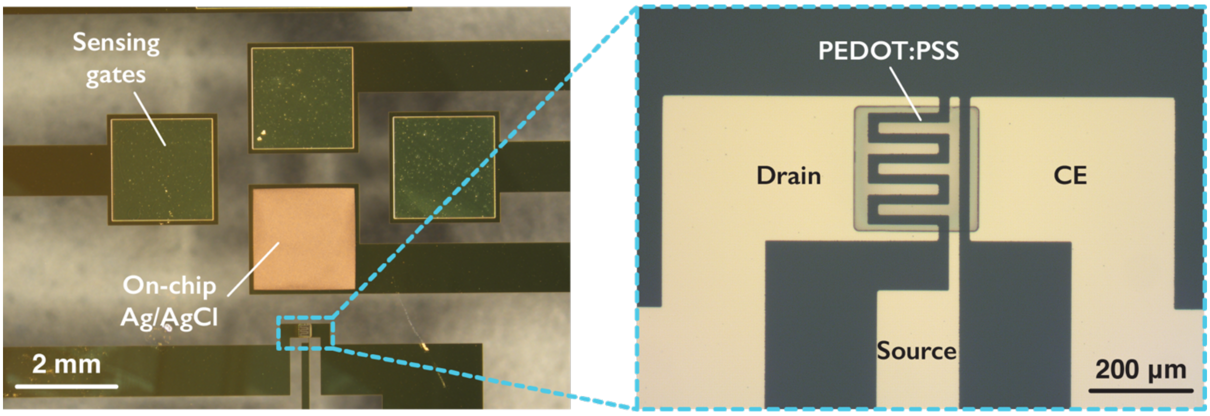

Supplementary Figure 2. Microscope image of the ref-OECT-based E-AB sensor. 3 working electrodes (sensing gates), an on-chip Ag/AgCl reference electrode and a PEDOT:PSS counter electrode (channel) with interdigitate drain and source electrodes are integrated together.

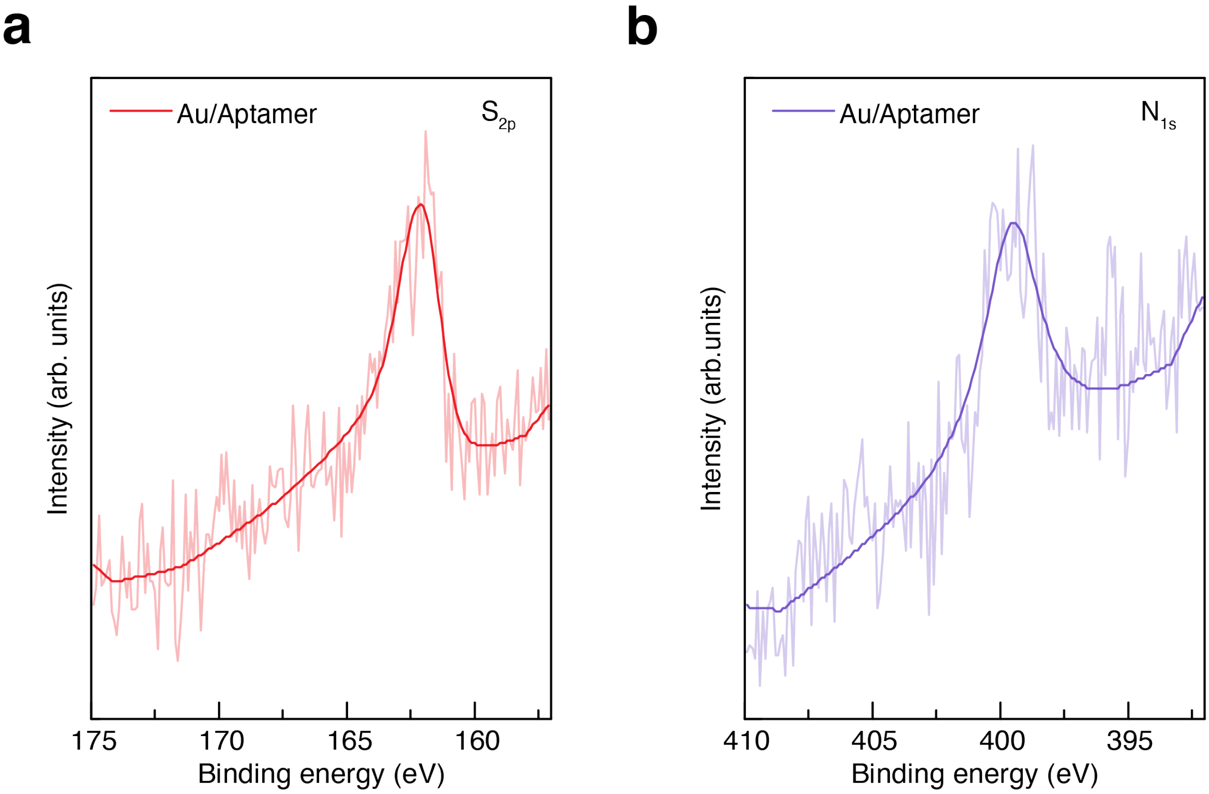

Supplementary Figure 3. XPS of modified electrode. (**a, b**) XPS of the S 2*p* and N 1*s* of the Au electrode after TGF-β_1 aptamer modification (after rinse by DI water).

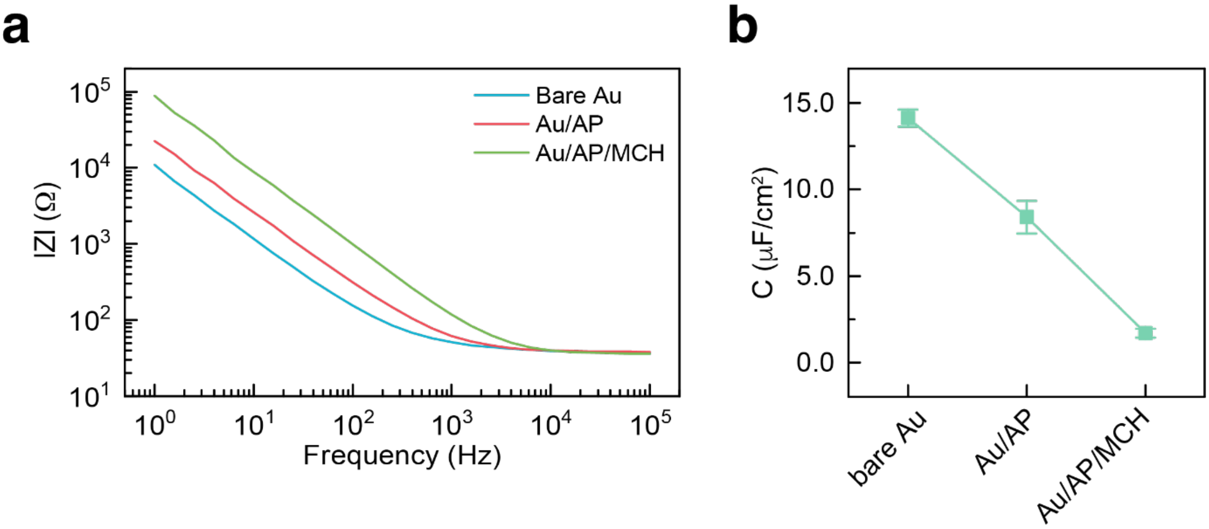

Supplementary Figure 4. EIS of modified electrode. (**a**) Comparison of the EIS results before and after aptamer/MCH modification. (**b**) Areal double layer capacitance of the Au electrode before and after aptamer/MCH modification from the fitting of the EIS data (error bars represent standard deviation, N=3).

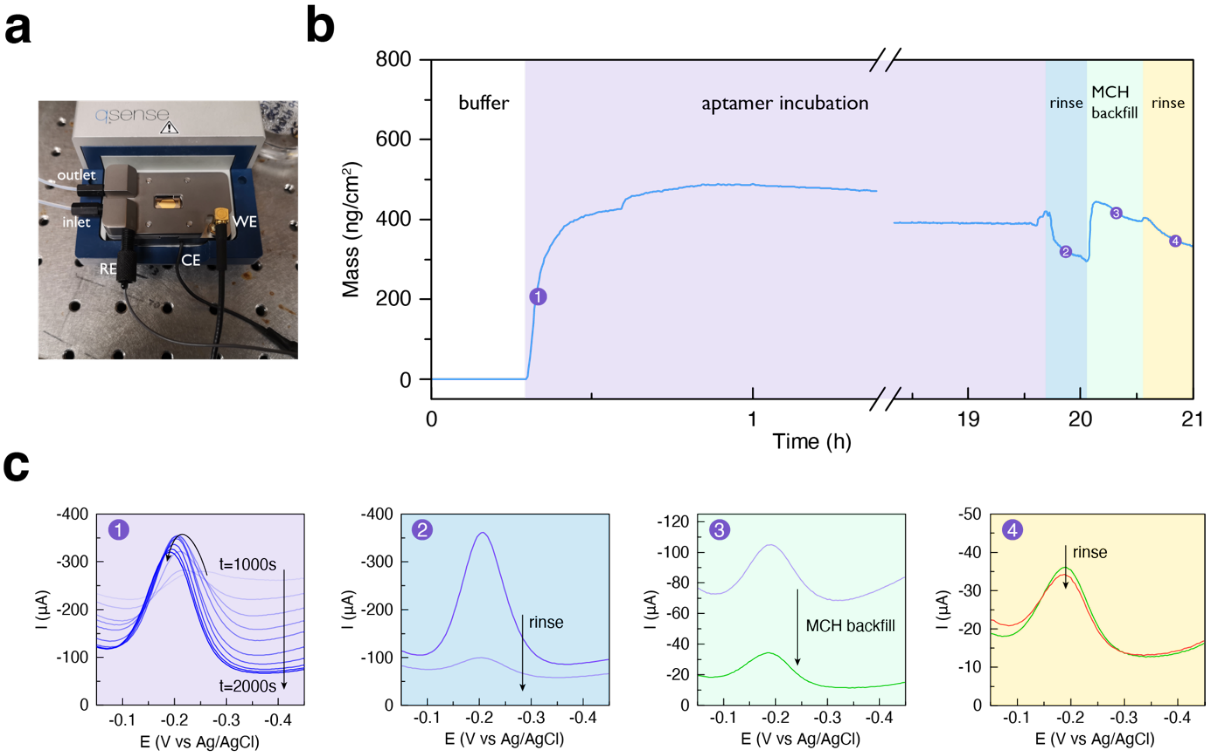

Supplementary Figure 5. Monitoring aptamer functionalization process with EQCM-D (**a**) Setup of EQCM-D for characterizing the aptamer modification. (**b**) Overall mass change on EQCM-D chip during the aptamer modification and MCH backfill. (**c**) SWV results of the EQCM-D chip during different stages: (1) introduction of aptamer; (2) rinse of aptamer; (3) MCH backfill; (4) rinse of MCH.

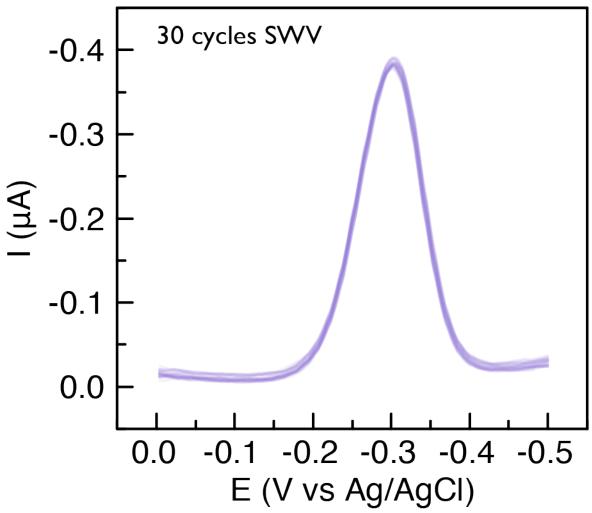

Supplementary Figure 6. Stability of aptamer modified Au electrode. 30 cycles of SWV scan overlapped for the aptamer modified Au electrode.

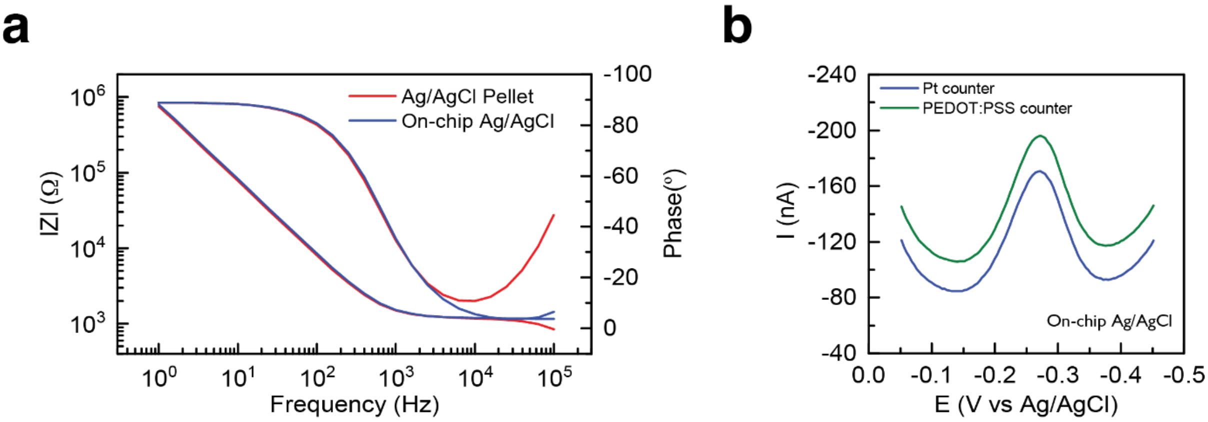

Supplementary Figure 7. Verification of the functionality of on-chip Ag/AgCl reference electrode and PEDOT:PSS counter electrode. (**a**) Comparison of the EIS results for an Au electrode with bulk Ag/AgCl pellet and on-chip Ag/AgCl reference electrode. (**b**) Comparison of the SWV results for an aptamer modified Au electrode with Pt or PEDOT:PSS counter electrode.

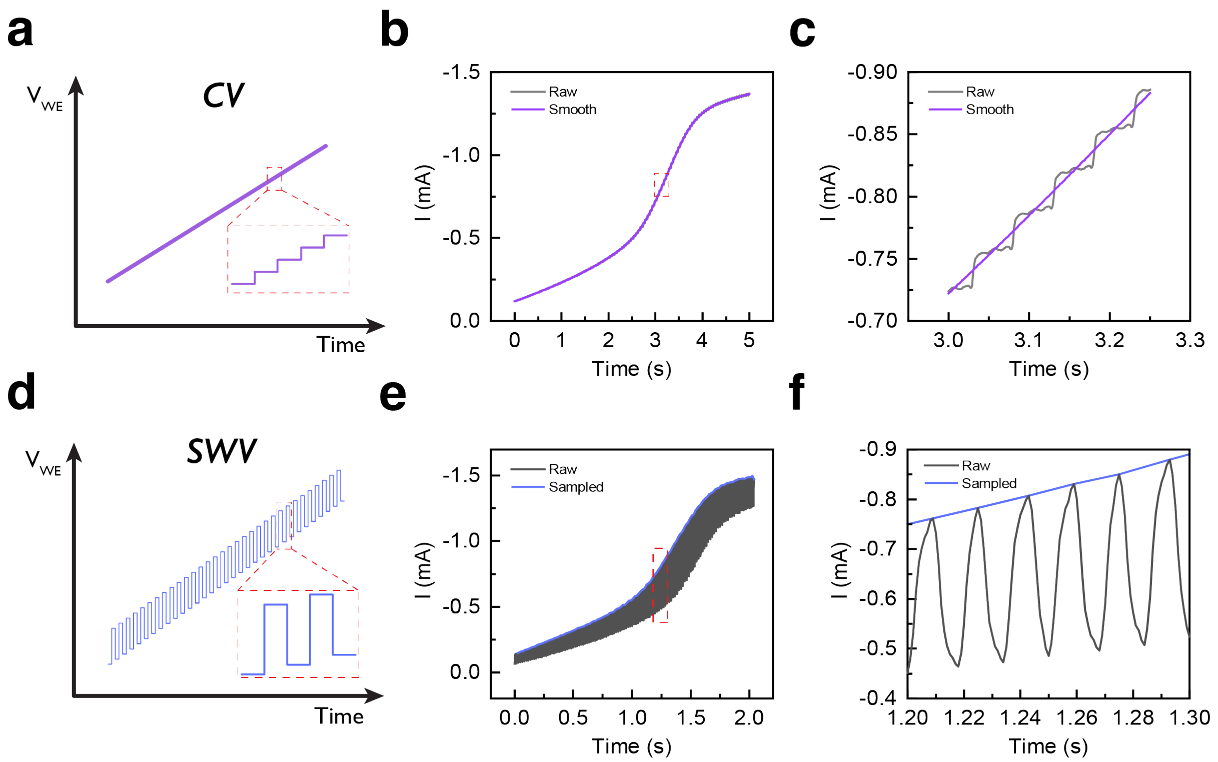

Supplementary Figure 8. Voltage waveform and current sampling strategy during operation. (**a**) Voltage waveform of CV measurement (voltage is ramped up stepwise instead of true linear). (**b, c**) Raw data and smoothed data of the channel current in ref-OECT-based E-AB sensor during CV-OECT operation. (**d**) Voltage waveform of SWV measurement. (**e, f**) Raw data and sampled data of the channel current in ref-OECT based E-AB sensor during SWV-OECT operation.

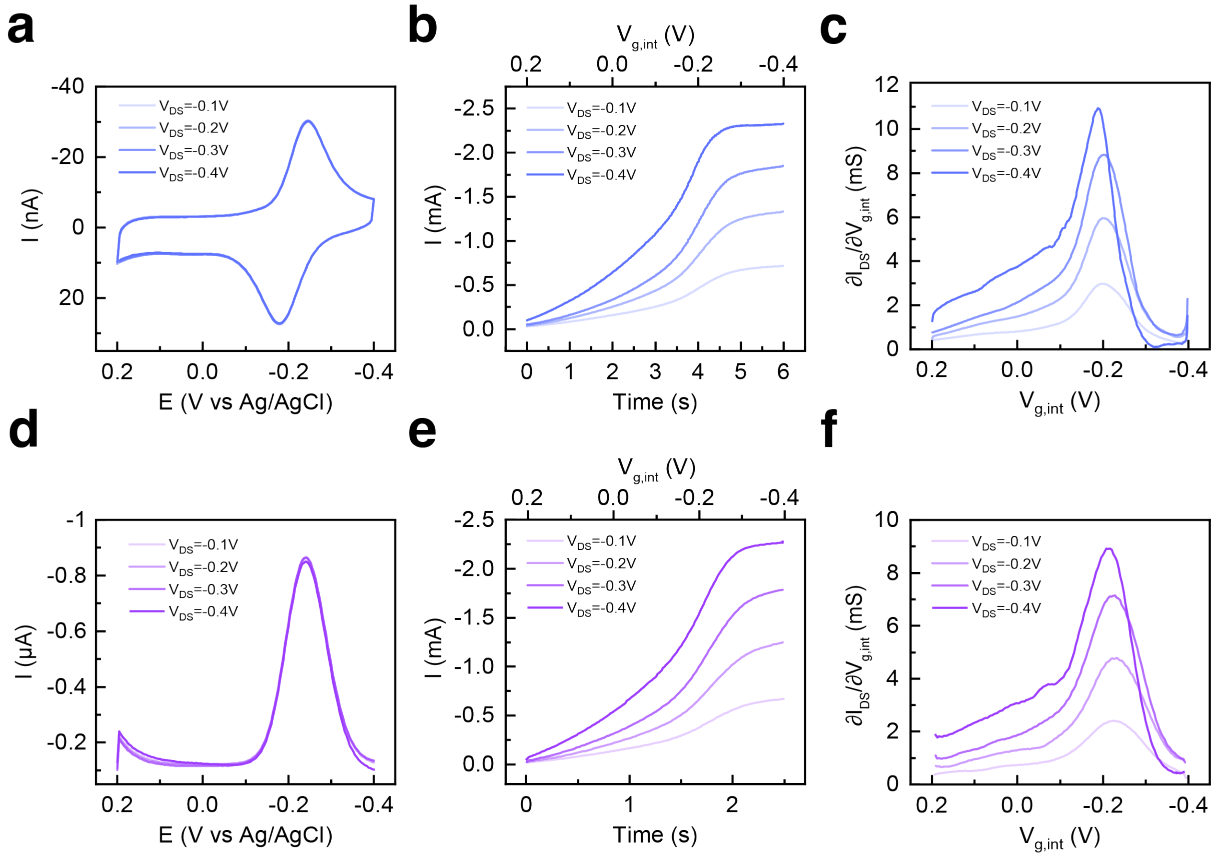

Supplementary Figure 9. Influence of V_{DS}. (**a, d**) CV and SWV results of the aptamer modified Au working electrode in bare 1xPBS buffer solution with different V_{DS}. (**b**, **e**) Channel current of ref-OECT based E-AB sensor in CV-OECT or SWV-OECT operation with different V_{DS}. (**c**, **f**) Slope of channel current of ref-OECT based E-AB sensor in CV-OECT or SWV-OECT operation with different V_{DS}.

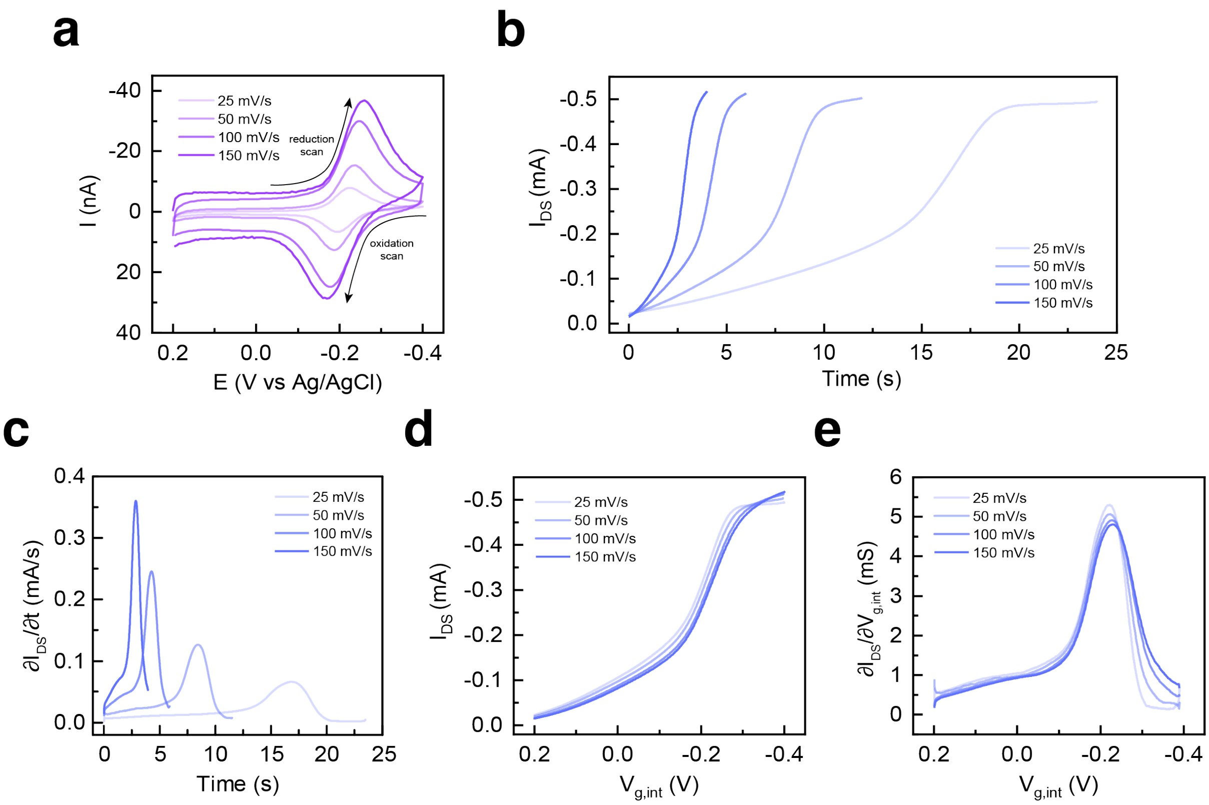

Supplementary Figure 10. Influence of scan rate. (**a**) Influence of scan rate of CV on the reduction and oxidation peaks of MB redox reporter in an aptamer modified Au working electrode. (**b, c**) Channel current and slope of channel current for ref-OECT with different scan rate when plotted as a function of time. (**d, e**) Channel current and slope of channel current for ref-OECT with different scan rate when plotted with respect to $V_{g,int}$. Only the channel current during reduction scan of MB redox reporter is shown here.

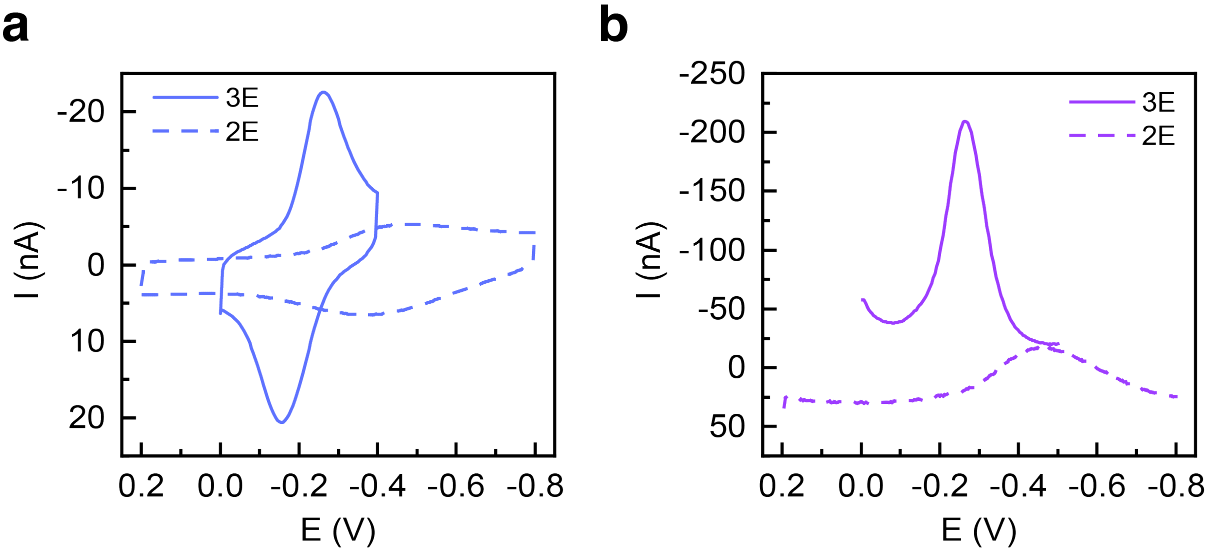

Supplementary Figure 11. Aptamer modified Au working electrode tested in both 3E and 2E setup. Comparison of the MB redox peak in 3E and 2E setup during (**a**) CV or (**b**) SWV measurement. For CV, the scan speed is 100 mV/s and the step size is 5 mV for both 2E and 3E setup. For SWV, the scan frequency is 60 Hz, pulse amplitude is 40 mV and step potential is 4 mV for both 2E and 3E setup. In all test, size of working electrode and reference electrode is 2 mm × 2 mm; the size of counter electrode is 240 µm × 240 µm.

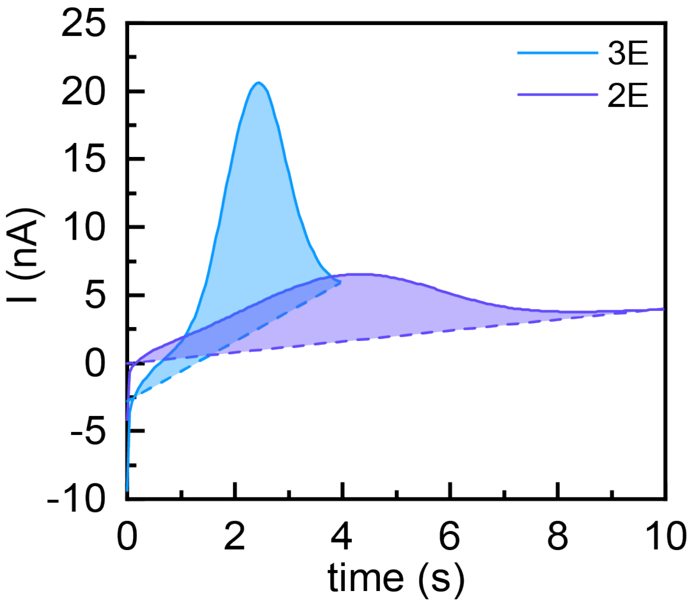

Supplementary Figure 12. Time integral of anodic peak of MB in both 3E and 2E testing setup. Oxidation peak area are comparable for 3E (27.4 nC) and 2E (22.5nC) after considering potential contribution from capacitive charging current.

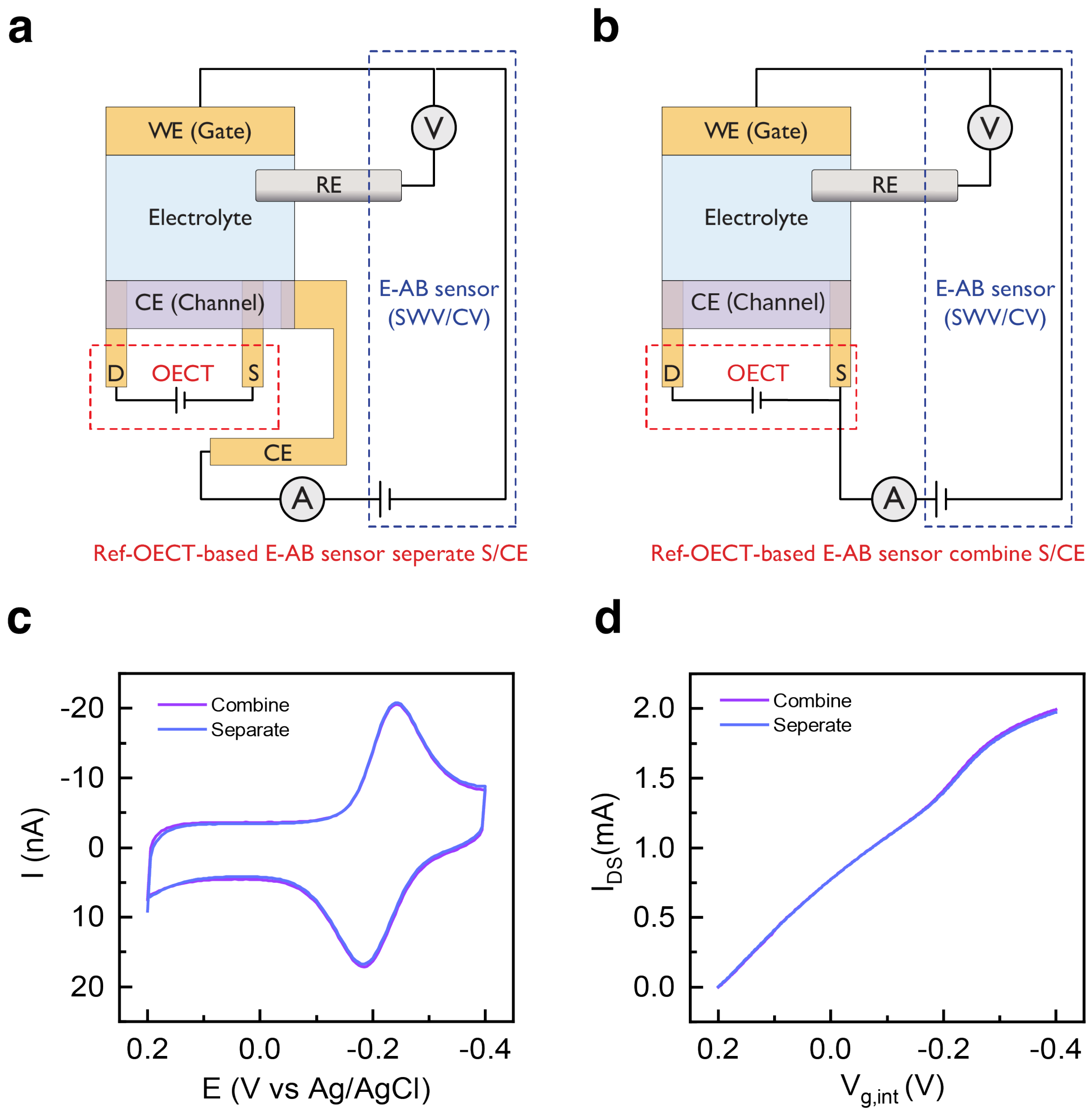

Supplementary Figure 13. Influence of testing scheme. Schematic image of the device testing scheme by either (a) separating the S and CE contact or (b) combining S and CE contact. (c) CV results in working electrode with two different testing schemes. (d) Channel current of OECT with two different testing schemes.

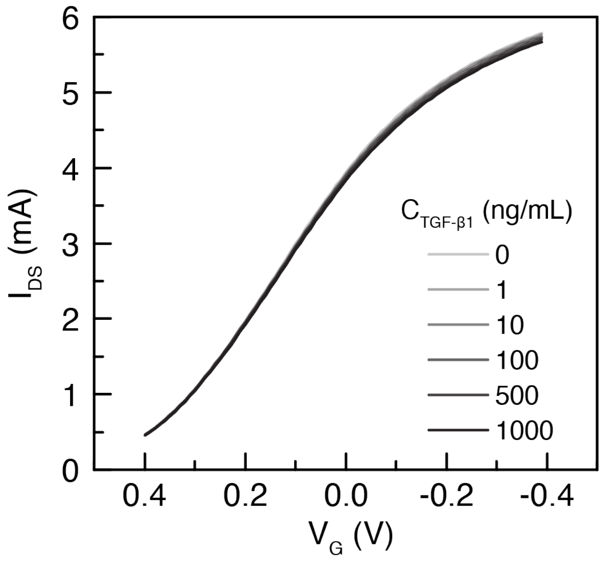

Supplementary Figure 14. Normal transfer curve measurement of OECT with PEDOT:PSS channel and on-chip Ag/AgCl gate during TGF-β$_1$ sensing. Negligible drift of transfer curves can be observed and confirm the stability of PEDOT:PSS channel.

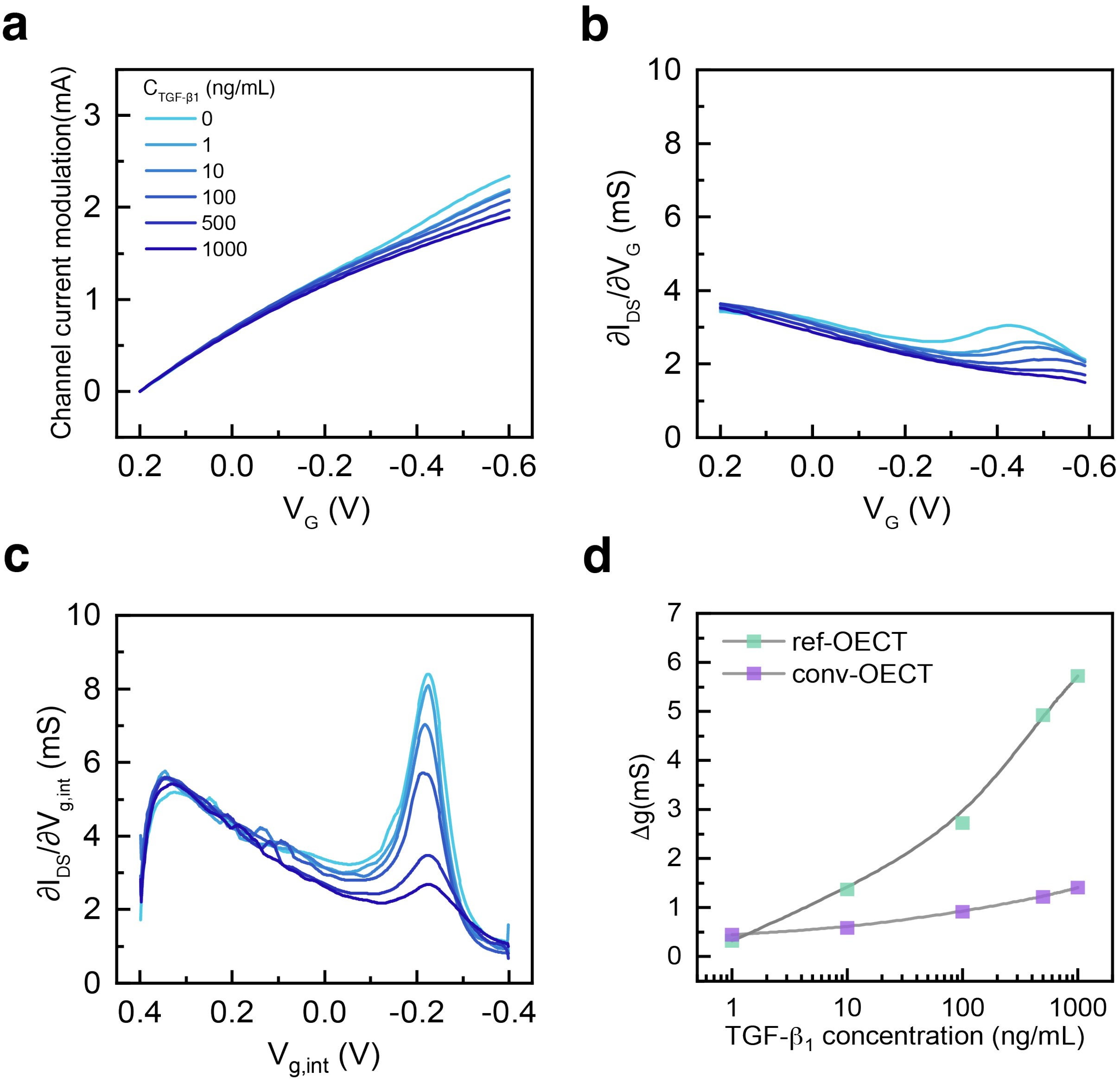

Supplementary Figure 15. TGF-β₁ sensing with conv-OECT-based E-AB sensor. (**a**) Transfer curves of conv-OECT-based E-AB sensor in electrolyte with different concentration of TGF-β_1. (**b**) Extracted transconductance of conv-OECT-based E-AB sensor with different TGF-β_1 concentration. (**c**) Extracted slope of channel current of ref-OECT-based E-AB sensor with different TGF-β_1 concentration. Original sensing results is from Figure 4b. (**d**) Comparison of the transconductance (conv-OECT)/slope of channel current (ref-OECT) with respect to the concentration of TGF-β_1. Ref-OECT based E-AB sensor shows a sensitivity of 2.90 mS/dec, which is around 6 times larger than that in conv-OECT based E-AB sensor (0.51 mS/dec).

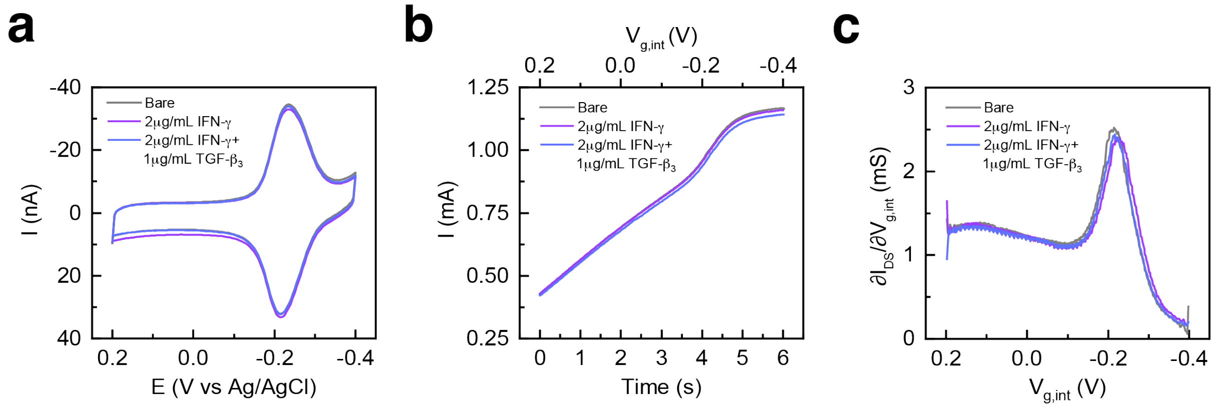

Supplementary Figure 16. Selectivity of ref-OECT-based E-AB sensor. (**a**) CV results of the aptamer modified Au working electrode in bare 1xPBS buffer solution and the PBS buffer solution with either 2μg/mL IFN-γ or 2μg/mL IFN-γ together with 1μg/mL TGF-β_3. (**b**, **c**) Channel current and slope of channel current of ref-OECT based E-AB sensor in bare 1xPBS buffer solution and the PBS buffer solution with either 2μg/mL IFN-γ or 2μg/mL IFN-γ together with 1μg/mL TGF-β_3.

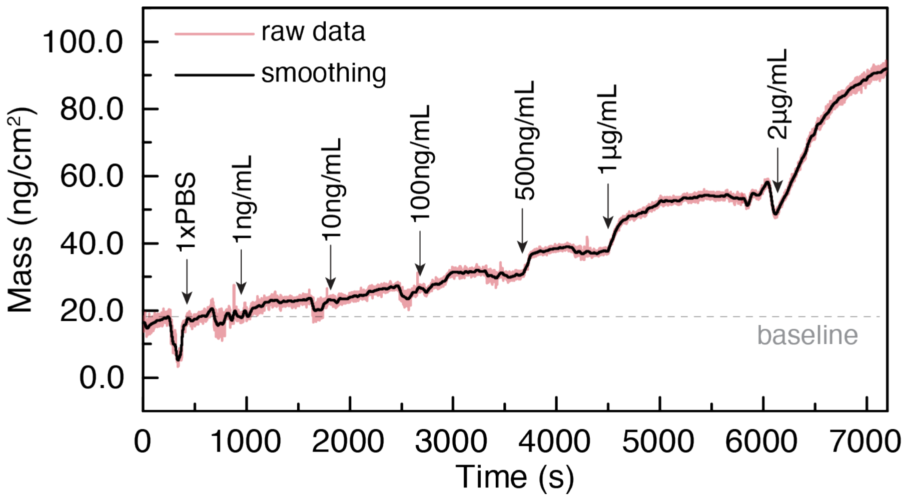

Supplementary Figure 17. Characterize aptamer TGF-β_1 binding with EQCM-D. Mass change of the aptamer modified EQCM-D chip after introducing TGF-β_1 with elevating concentration.

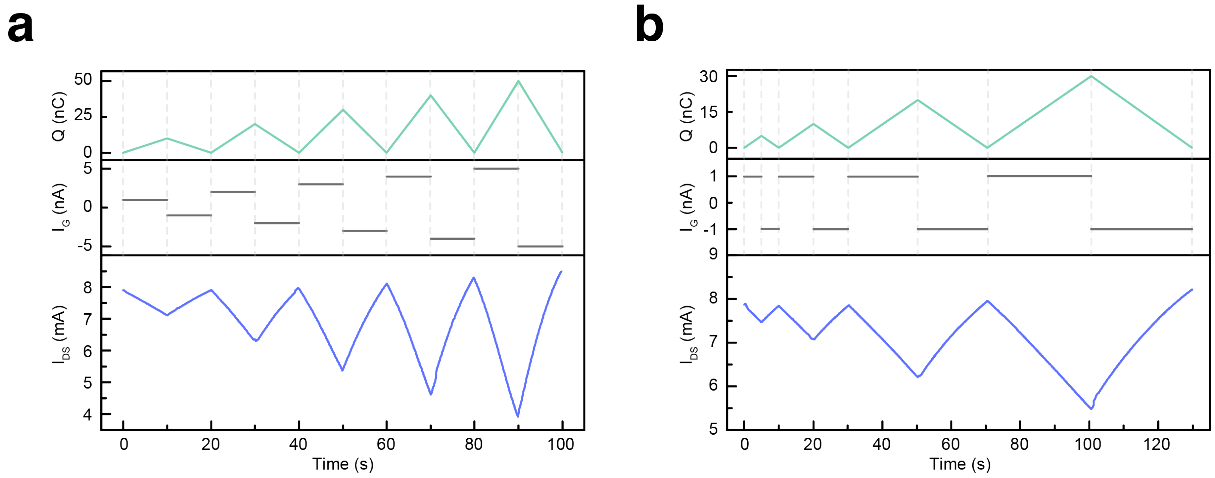

Supplementary Figure 18. Operation of OECT with constant gate current. (**a**) OECT channel current change with different constant gate current ($\pm 1\ nA, \pm 2\ nA, \pm 3\ nA, \pm 4\ nA, \pm 5\ nA$) for 10s. (**b**) OECT channel current change with $\pm 1\ nA$ gate current for different time (5s, 10s, 20s, 30s).

Supplementary References

1 Matharu, Z. *et al.* Detecting transforming growth factor-β release from liver cells using an aptasensor integrated with microfluidics. *Anal. Chem.* **86**, 8865-8872 (2014).

2 Zhou, Q. *et al.* Liver injury-on-a-chip: microfluidic co-cultures with integrated biosensors for monitoring liver cell signaling during injury. *Lab on a Chip* **15**, 4467-4478 (2015).

3 Gao, Y. *et al.* A flexible multiplexed immunosensor for point-of-care in situ wound monitoring. *Science Advances* **7**, eabg9614 (2021).

4 Zhou, L. *et al.* An extracellular matrix biosensing mimetic for evaluating cathepsin as a host target for COVID-19. *Anal. Chim. Acta* **1225**, 340267 (2022).

5 Sánchez-Tirado, E. *et al.* Viologen-functionalized single-walled carbon nanotubes as carrier nanotags for electrochemical immunosensing. Application to TGF-β1 cytokine. *Biosensors and Bioelectronics* **98**, 240-247 (2017).

6 Yao, Y. *et al.* Biomarkers of liver fibrosis detecting with electrochemical immunosensor on clinical serum. *Sensors Actuators B: Chem.* **222**, 127-132 (2016).

7 Sánchez-Tirado, E., González-Cortés, A., Yáñez-Sedeño, P. & Pingarrón, J. Carbon nanotubes functionalized by click chemistry as scaffolds for the preparation of electrochemical immunosensors. Application to the determination of TGF-beta 1 cytokine. *Analyst* **141**, 5730-5737 (2016).
